# Alignment of Continuous Auditory and Visual Distractor Stimuli Is Leading to an Increased Performance

**DOI:** 10.3389/fpsyg.2020.00790

**Published:** 2020-05-08

**Authors:** Stefanie Mühlberg, Matthias M. Müller

**Affiliations:** Institute of Psychology, University of Leipzig, Leipzig, Germany

**Keywords:** cross-modal, attention, bottom–up, vision, audition

## Abstract

Information across different senses can affect our behavior in both positive and negative ways. Stimuli aligned with a target stimulus can lead to improved behavioral performances, while competing, transient stimuli often negatively affect our task performance. But what about subtle changes in task-irrelevant multisensory stimuli? Within this experiment we tested the effect of the alignment of subtle auditory and visual distractor stimuli on the performance of detection and discrimination tasks respectively. Participants performed either a detection or a discrimination task on a centrally presented Gabor patch, while being simultaneously subjected to a random dot kinematogram, which alternated its color from green to red with a frequency of 7.5 Hz and a continuous tone, which was either a frequency modulated pure tone for the audiovisual congruent and incongruent conditions or white noise for the visual control condition. While the modulation frequency of the pure tone initially differed from the modulation frequency of the random dot kinematogram, the modulation frequencies of both stimuli could align after a variable delay, and we measured accuracy and reaction times around the possible alignment time. We found increases in accuracy for the audiovisual congruent condition suggesting subtle alignments of multisensory background stimuli can increase performance on the current task.

## Introduction

In our inherently multisensory world, the selection and integration of information within and across senses is the foundation of perception. This role is fulfilled by attention, which selects relevant information from the stream of sensory information to be processed (Talsma et al., 2010; Macaluso et al., 2016) via two distinctive processes: a voluntary allocation of attention and an involuntary capture of attention. Voluntary or top–down attentional processes are generally driven by our behavioral goals (Spence and Driver, 2004; Macaluso, 2010). Involuntary or bottom–up attentional processes are driven by the properties of the stimulus itself (Hopfinger and West, 2006). They are reflexive, resulting in an automatic involuntary attraction of attention to a salient event, which is called “pop out effect” (Duncan and Humphreys, 1989; Katsuki and Constantinidis, 2014; Tang et al., 2016).

Similar to unisensory space, such interactions had also been found in multisensory contexts. In a prominent study, Van der Burg et al. (2008) presented brief auditory stimuli which temporally coincided with the target of a visual search display and found that the temporal alignment of the auditory stimuli led to a “pop-out” of the visual target, i.e., a decreases in time needed to complete the visual search task. This result has since been repeatedly replicated (Matusz and Eimer, 2011; Van der Burg et al., 2011), even with other stimulus combinations, such as vision and touch (Van der Burg et al., 2009; Ngo and Spence, 2010) or even vision and olfaction (Chen et al., 2013). However, the stimulus enabling the target to pop had to be transient in nature (Van der Burg et al., 2010) and it required to be spatially (Santangelo and Spence, 2007; Spence and Santangelo, 2009; Santangelo et al., 2013) or temporally aligned with the target (Van der Burg et al., 2008, 2009). But how do stimuli that are not spatially and temporally aligned with the target affect our performance? A common observation is that unrelated events negatively affect task performances, i.e., generating behavioral costs (Parmentier, 2008; Parmentier et al., 2010, 2011, 2019), although the synchronization of distractors can also speed up responses toward upcoming events (Van der Burg et al., 2008). Event-related potential (ERP) studies show that these distraction effects occur relatively early in cortical stimulus processing, by modulating the amplitude of the N2 and P3 ERP amplitudes (Bendixen et al., 2010; Demeter et al., 2016). In all these studies however, the stimulus is novel or very transient with a sudden onset, thus they cannot answer the question in how far a multisensory alignment of task irrelevant stimuli itself can capture attention and serve as a distractor.

Within this study, we tried to identify if the alignment of two continuous distractor stimuli, one visual and one auditory, would lead to an increased or decreased behavior performance. Considering that the alignment of distractor stimuli does not share any spatial or temporal properties with the target stimulus we hypothesize that the multisensory stimulus would serve as a distractor, decreasing the task performance and we are measuring the reaction times, accuracy and the time scale of the effect. Participants performed a detection or discrimination task on a centrally presented Gabor stimulus, while simultaneously being exposed to a moving random dot kinematogram and a binaural tone. Both the visual and the auditory distractor were frequency modulated; the visual distractor through an isoluminant color change and the auditory distractor through an amplitude modulation. While the frequency is initially different for both competing stimuli, the sound changes its modulation frequency after a variable time delay to either match the frequency of the visual stimulus (synchronized) or to switch to a different frequency that does not match the frequency of the visual stimulus (unsynchronized). In addition, we presented a visual control condition, which utilized the same visual distractor as in the audiovisual conditions, but instead of a frequency modulated tone we presented continuous white noise which would allow us to analyze the impact of the auditory tone switch itself upon our pattern of results. We measured accuracy and reaction times around the time of the auditory frequency change in order to identify if multisensory alignment or synchronization affects task performance. In order to uncover the temporal dynamics of such an effect, target events at the centrally displayed task stimulus were presented at different time points after the auditory frequency change.

## Methods

### Participants

Twenty four participants (two male, age range 18–32 years, 21 right handed) with normal or corrected to normal vision and hearing participated in the study. Participants were reimbursed with course credits or 8€ per hour and gave written and informed consent in advance of the experiment. The sample size of the experiment was calculated *a priori*, using G-Power (Erdfelder et al., 1996; Faul et al., 2007), on the basis of the effect sizes from Van der Burg et al. (2008). Comparable significant behavioral modulations (α = 0.05, η^2^=0.68) for our condition effect (visual, auditory, audiovisual congruent and audiovisual incongruent) can be detected with 90% power using 24 Participants.

### Stimulus Design

**FIGURE 1:**
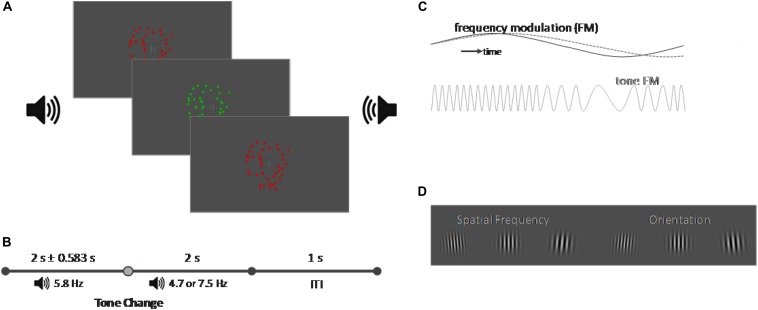
Schematic representation of the task (not in scale). **(A)** Schematic representation of the experimental setup. A central Gabor patch was presented on a gray screen. The Gabor patch was surrounded by a random dot kinematogram which changed its color from red to green with a frequency of 7.5 Hz. A binaural tone was presented via two loudspeakers on the left and right side of the screen which amplitude was manipulated with a frequency of 5.8 Hz [frequency manipulation (FM)]. **(B)** Timeline of a trial. Each trial started with the onset of the sound and of the random dot kinematogram. While the initial FM frequency of the tone was always 5.8 Hz, the FM frequency would change after a variable delay of 2 ± 0.583 s the FM frequency and either align with the frequency of the color change of the random dot kinematogram (7.5 Hz) or to an unrelated frequency (4.7 Hz). The trial ended 2 s after the tone change and after another second the next trial started. **(C)** FM representation. If the FM of the random dot kinematogram and of the tone were aligned, it created an audiovisual color pulse, in contrast to an unaligned tone and the random dot kinematogram dashed gray line. **(D)** Representation of the target events which changed both their spatial frequency and orientation. Participants performed either a detection task on one feature or a discrimination task which required responding to two combinations of the features.

Visual Stimuli were presented upon a screen with a 1920 × 1080 pixel resolution and a 120 Hz refresh rate via a PROPixx DLP color projector (VPixx Technologies, Inc., Saint-Bruno, QC, Canada). Upon a gray background a centrally located Gabor patch with a diameter of 6.64° visual angle (see Figure 1A) was presented throughout the whole course of the experiment.

The experiment consists of three different conditions: an audiovisual congruent condition, an audiovisual incongruent condition and a visual control condition. All conditions each presented one visual and one auditory distractor stimulus. The visual distractor stimulus was identical in all three conditions and consisted of a random dot kinematogram with a diameter of 10° visual angle and a movement speed of 20 pixel per second. The color of the random dot kinematogram was modulated and changed from red to green with a frequency of 7.5 Hz. The auditory distractor stimulus was a continuous tone, presented binaurally over loudspeakers positioned on both sides of the screen. Each condition presented a different type of tone. For the visual condition we presented continuous white noise. In the audiovisual congruent condition, we presented a tone with a center frequency of 440 Hz whose pitch was modulated sinusoidally by 10% (± 44 Hz) with an initial frequency of 5.8 Hz [Figure 1B, frequency modulation (FM)]. After 2 s ± a jitter of up to 583 ms the frequency of the FM modulation would switch to 7.5 Hz to align with the modulation of the visual stimulus. In the audiovisual incongruent condition, we presented the same initial FM modulated tone, but it’s frequency would switch to 4.7 Hz instead. For the random dot kinematogram we presented a large circle (8° visual angle) in the center of the screen which flickered either red or green with a frequency of 8 Hz. Participants had to adjust the luminance of the color until the flicker seem to vanish against the background, which would be the point at which they perceive the color as isoluminant against the background. Auditory stimuli were adjusted to 35 dB above the individual sensation level (SL), recorded through the method of limits (Leek, 2001). The goal of these adjustments of the visual and auditory distractor stimuli was to make their FM subtle or, in other words, minimally distracting for the participant on their own, as we predicted that the alignment of the FM frequency would lead to a multisensory alignment and increase the saliency of the distractors without the onset of a transient event. Distractor stimuli were chosen to allow for an adjustment of the experiment toward EEG or different distractor natures. Their onset was set a minimum of 500 ms before the first possible onset of a target and presented continuously, so any responses toward the target should not be caused by transient onsets or changes in the distractor stimuli. In addition, the random dot kinematogram would also allow the modulation of movement speed, coherence and direction as modulated features, and a modulation of isoluminant color was chosen to provide a distractor stimulus that should not capture exogenous attention on its own, as there were no changes in the overall physical properties of the random dot kinematogram.

### Experimental Design and Procedure

Participants were seated comfortably in an electromagnetically shielded and acoustically dampened recording chamber with their gaze directed frontally to the screen at 115 cm distance.

An overview of the trial structure is given in Figure 1C. Each trial started with the presentation of the visual and auditory distractor stimuli. A tone change occurred within the audiovisual congruent and incongruent conditions after 2 s ± a variable jitter of up to 583 ms; the white noise in the visual condition would remain unaltered.

Each trial contained a minimum of one target event consisting of a simultaneous change of the spatial frequency and orientation of the centrally presented Gabor stimulus. Participants had to perform either a detection task upon the change in spatial frequency or orientation of the Gabor, or a discrimination task, where they were instructed to only respond to two specific combinations of the spatial frequency and orientation, such as an increase in spatial frequency paired with a left tilt of the Gabor stimulus (Figure 1D). We utilized a Block design; with the blocks alternating in an AB fashion, and the starting block, as well as the to-be-attended feature was counterbalanced. The difficulty of the two tasks was adjusted for each participant individually in advance of the experiment. For this, we designed a short training, consisting of 20 trials, in which the participants were instructed to perform the detection task on their respective feature with an orientation change of 7° and a change of spatial frequency of 0.015° of visual angle. No distractor stimuli were presented in the training session. If participants performed the task correctly between 85 and 95%, the value for the respective feature change was saved, else the value would be either increased or decreased by 2° (orientation) or 0.003° (frequency) and the training repeated until participants could perform it with an accuracy between 85 and 95%. In a second step, the difficulty for the discrimination task was adjusted. Taking the value of the previously adjusted feature, the remaining feature was adjusted by the same steps until a discrimination performance between 60 and 70% was reached.

We were interested in how the change of the auditory FM frequency was influencing the behavioral performance of the participants and to determine this we manipulated the onset of the target event in relation to the FM change of the tone. If trials contained one target event, we placed the target event within 1 of 10 equally sized time windows (58.3 ms each) starting from −58.3 ms (0 being the time of the FM switch) and 524.7 ms. Trials containing two target events had an additional early event, which could appear from 500 ms after the trial onset, and which had a minimum distance of 700 ms from the second target event of trial. 2/3 of all trials of the experiment contained one target event, while in the remaining 1/3 of the trials a second target event was presented and participants were instructed to respond to all target events. Each time window contained an equal number of the target events over the course of the experiment; similarly, the remaining periods before and after the fine tuned time windows contained an equal number of events.

The experiment consisted in total of 900 pseudorandomized trials, 1/3 of them belonging to the visual condition, 1/3 to the audiovisual congruent condition and 1/3 to the audiovisual incongruent condition. This means that we had 10 trials per condition (visual, audiovisual congruent, audiovisual incongruent) per time window. The trials were organized in 10 blocks of 90-trial length each, with an average block duration of 5 min.

### Data Analysis

We classified correct answers as correct identifications or rejections of target events given between 200 and 900 ms, and calculated the accuracy by dividing the sum of the numbers of hits and correct rejections by the sum of the all targets and distractors. In addition, we recorded the mean reaction time for each condition and participant.

Data were subjected to a three-way repeated measures analysis of variance (ANOVA) with the factors of *task* (detection vs. discrimination), *condition* (visual, audiovisual congruent, or audiovisual incongruent), and *time* [11 possible onsets, early (more than 58.3 ms before the tone change) : −58.3 to 0, 0 to 58.3, 58.3 to 116.6, 116.6 to 174.9, 174.9 to 233.2, 233.2 to 291.5, 291.5 to 349.8, 349.8 to 408.1, 408.1 to 466.4 and 466.1 to 524.7 ms]. Effect sizes are given as eta-squared (η^2^) and, when applicable, a Greenhouse–Geisser correction of the degrees of freedom was applied to control for violations of the assumption of sphericity (Greenhouse and Geisser, 1959).

When necessary, two-tailed paired *t*-tests had been applied for *post hoc* testing, and the *p*-values were corrected for multiple comparisons using the Bonferroni–Holm procedure (Holm, 1979).

## Results

### Accuracy

Participants could perform both tasks with an above-chance accuracy of 0.9112 (*SD* = 0.1503) for the detection task and 0.7768 (*SD* = 0.1907) for the discrimination task. The accuracies are higher than in the training because participants improved over the course of the experiment, but all reported that they perceived the discrimination task as very demanding.

**FIGURE 2:**
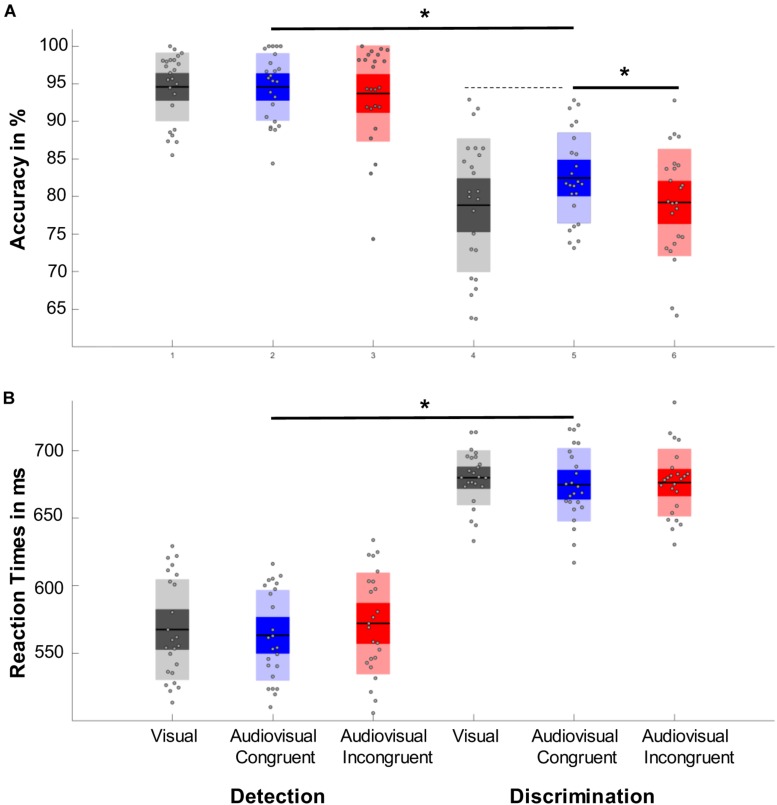
Representation of the main effects of task and condition. Significant results are marked with an asterisk, while dashed lines represent marginal significant effects. **(A)** Accuracy results. Responses to the detection task were significantly more accurate than responses in the discrimination task. One can also observe an effect of condition with responses in the audiovisual congruent condition (blue) were significantly more accurate than responses in audiovisual incongruent trials (red) and marginally more accurate than responses toward the visual control condition (gray). **(B)** Reaction time results. While participants responded significantly faster in the detection trials no other effect reached significance.

**FIGURE 3:**
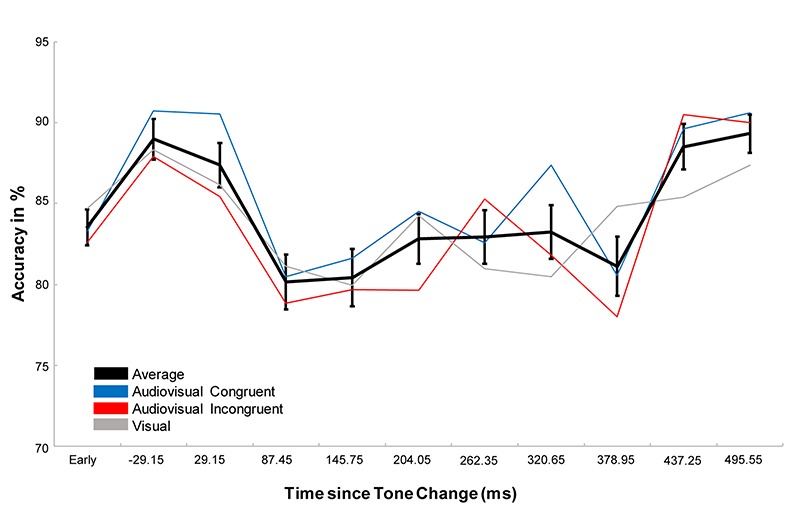
Representation of the effect of time. The black line represents the average over all conditions, the blue and red line the congruent and incongruent conditions respectively and the gray line the visual control. Error bars display the standard error of the mean. While the accuracy after the tone change initially increases, accuracy starts to drop between 58.3 and 116.6 ms after the tone change and stay lower than during the early window until about 466.1–524.7 ms after the tone change. While the accuracy drop is significant against the accuracy directly after the tone change, neither the initial increase, nor the following drop and recovery is significant against the early baseline window. Interestingly, one can observe stronger increases and reduced drops in the audiovisual congruent conditions, but the effect is not significant.

Performing a repeated measure ANOVA we found a main effect of task (*F*_1,23_ = 64,202, *p* < 0.001, η^2^ = 0.736), which revealed that participants performed more accurately in the detection task than in the discrimination task. We also found a main effect of condition (*F*_1.764,40.561_ = 4.427, *p* = 0.017, η^2^ = 0.161) with a significantly higher accuracy in the audiovisual congruent condition compared to the audiovisual incongruent condition (*p* = 0.023) and a marginally higher accuracy than the visual condition (*p* = 0.078, Figure 2A). In addition, we found a main effect of time (*F*_3.007,69.172_=5.777, *p* < 0.001, η^2^ = 0.201), which revealed that the accuracy first increased after the tone change, only to then significantly drop for the next 350 ms and then to rebounce (Figure 3). However, every time window failed to reach significance against the early baseline window. No other effect reached significance; we did however, observe a marginal interaction between condition and time (*F*_9.517,218.889_=1.488, *p* = 0.080, η^2^ = 0.061), suggesting that the initial accuracy increase is potentially more pronounced in the audiovisual congruent condition, and a marginal interaction between condition and task (*F*_1.957,45.027_=2.640, *p* = 0.082, η^2^ = 0.102), revealing marginally stronger condition differences in the discrimination task. The interaction between time and task (*F*_6_._449,148_._348_=0.583, *p* = 0.826, η^2^ = 0.024) and between time, condition and task (*F*_9.007,208,773_=0.757, *p* = 0.765, η^2^ = 0.031) remained clearly insignificant.

### Mean Reaction Times

The average reaction time for the detection task was 569.97 ms (*SD* = 60.41 ms) and 675.22 ms for the discrimination task (*SD* = 58.41 ms).

A repeated measure ANOVA revealed a main effect of task (*F*_1,23_=195.197, *p* < 0.001, η^2^ = 0.895) with faster responses in the detection task (Figure 2B). In addition, we found a marginal main effect of time (*F*_0.698,160,721_ = 1,790, *p* = 0.633, η^2^ = 0.827), with a slight decrease in reaction time the first 300 ms after the tone change. The main effect of condition (*F*_1.907,43.878_=195.197, *p* = 0.423, η^2^ = 0.191), as well as the interactions between task and condition (*F*_1_._789,41.161_=195.197, *p* = 0.400, η^2^ = 0.201), condition and time (*F*_9.400,216,201_=195.197, *p* = 0.769, η^2^ = 0.594), time and task (*F*_5.965,137.204_=195.197, *p* = 0.142, η^2^ = 0.737) and their three-way interaction (*F*_9.780,224.946_=195.197, *p* < 0.821, η^2^ = 0.557) remained insignificant.

### Supplementary – Addition of an Auditory Control

Within a pilot study we tested the paradigm with a detection task upon the fixation cross on 23 participants. An image of the task is presented in Supplementary Figure 1.

The experiment had three main differences. First, we utilized a different task, since the results of the pilot differed greatly between participants. Second, each time window had a length of 150 ms, leading to a lower temporal resolution than in the main experiment. This helped us to identify the approximate time frame of interest for the main experiment and to increase the temporal resolution of the main experiment. And last, we added an auditory control in which the random dot kinematogram was still moving, but its color stayed a constant red. The control was important in order to identify any transient effect related to the change of the tone itself. However, while we found a main effect of condition (*F*_2.44,53.77_ = 3.660, *p* = 0.016, η^2^ = 0.143), all effects were independent of the tone itself, since the auditory conditions showed no significant difference either between each other or when compared to the audiovisual and visual conditions (all *p*-values > 0.1). An overview of the results can be seen in Supplementary Figure 2 and a timeline for all conditions is given in Supplementary Figure 3.

## Discussion

We investigated the effects of subtle multisensory alignment upon focused attention by presenting two continuously modulated distractor stimuli that could align their FM frequency after a variable delay. Our results revealed that a multisensory alignment of the two distractor stimuli increased the task accuracy in detection and discrimination tasks. This increase was significant against the audiovisual incongruent condition, but only marginally significant against the visual control.

Distractor stimuli often lead to a decreased performance (Parmentier, 2008; Bendixen et al., 2010; Parmentier et al., 2010, 2019; Demeter et al., 2016) due to increase in reaction time (Parmentier et al., 2010, 2019), while there is evidence for both an increase (SanMiguel et al., 2010; Marsja et al., 2018) and a decrease in accuracy (Demeter et al., 2016). In contrast to the majority of these studies the multisensory alignment of our auditory and visual events led to no significant modification of reaction times, but an increase in accuracy. One reason why could be that in most of the studies the authors used brief, transient events, such as flashes or brief sounds (Escera et al., 2003; Parmentier, 2008), leading to lower accuracy and increased reaction times (Bendixen et al., 2010; Parmentier et al., 2010), while we utilized a non-transient alignment of the auditory and visual distractors. An attentional capture through distractor stimuli can be explained by novelty (Parmentier, 2008; Parmentier et al., 2011, 2019), albeit also the informational value of the distractor is of importance, indicating that distractor encoding for information about the target can facilitate behavioral performance (Parmentier et al., 2010; Schomaker and Meeter, 2014; Klein, 2018). Such a facilitation could be compared to the pip and pop effect observed by Van der Burg et al. (2008). In contrast to these studies, we used subtle auditory and visual events, which only after a variable time interval could align in their modulation frequency and likely be integrated to a common percept. The alignment of the auditory and visual events was also independent of the onset of the target events, in contrast to the sounds used by Van der Burg (Van der Burg et al., 2008). As such, the stimulus is not novel, but presented continuously over the course of the trial, which does not lead to a pip and pop effect either (Fujisaki et al., 2006; Van der Burg et al., 2010). However, in contrast to an ongoing distractor, our stimuli can realign in their frequency in time, which we hypothesized to lead to multisensory integration. The signal elicited by a multisensory event is greater than the sum of the underlying unisensory signals (Stein and Stanford, 2008); thus, the multisensory stimulus should be more salient than the unisensory ones, and should be able to capture attention without a physical transient component. Since we only found an absolute increase for the audiovisual synchronous condition, one can assume that participants were integrating the signal and that it affected their perception, albeit not as a distractor.

One might wonder to what extent our results are merely the result of an unspecific speed-up of the auditory FM frequency in the audiovisual condition. Increases of stimuli sizes have been previously shown to increase sensitivity levels in behavioral tasks (Corbetta et al., 1991), and more frequent presentations of auditory stimuli do also increase the fMRI signal strength (Rinne et al., 2005). However, some evidence suggests that auditory FM modulations peak before 8 Hz (Zwicker, 1952) and that FM fluctuation strengths peaks at 4 Hz (Fastl, 1983). Since our modulation frequency changes from 5.8 to 7.5 Hz in the congruent condition and from 5.8 to 4.7 Hz in the incongruent condition one would have to assume that the switch in the incongruent condition should lead to a higher fluctuation strength and ultimately to a more salient event. Nonetheless, we do not observe a significant difference between the audiovisual incongruent condition and the visual control condition, which could mean that the auditory switch itself is not responsible for the pattern of results. Support for this hypothesis comes from the results of the supplementary experiment, which found no differences between the auditory control condition and any of the other conditions. If, however, the direction of the FM synchronization matters, then we should have observed significant difference between the auditory control and the visual and audiovisual incongruent conditions. Ultimately, however, we did not control for direction of the FM change in the main experiment, a possible caveat that it would be interesting to address in a future experiment.

It is possible that our audiovisual synchronous stimulus served neither as a distractor, as in the studies of Parmentier et al. (2010, 2011, 2019), nor as a facilitating stimulus, as in Van der Burgs studies (2008, 2010, 2013). Similarly, the alignment cannot have served as an alertness signal, since the visual behavioral results of the visual condition seem to lie between the audiovisual congruent and incongruent condition; however, since there was no auditory change, nothing could have served as an intermediate alertness signal in the visual condition.

Possibly the alignment of the visual and auditory distractor stimuli led to an increase in the stochastic resonance. Gleiss and Kayser (2014) performed a visual contrast detection task in which participants were subjected either to target synchronized clicks or to continuous white noise. Calculating psychometric curves, they found comparable shifts of both psychometric curves, which could reflect a general improvement through a cross-modal presentation of an event. The effect was attributed to an increase in the stochastic resonance. Support for this theory comes from Méndez-Balbuena et al. (2015). The authors used a visual pattern reversal task and measured the visual evoked response, while introducing additional tactile noise and found an increase in the P100 amplitude through the noise. Mizukami et al. (2018) used a somatosensory Go/No-Go task and investigated the effect of white noise on ERP components and found amplified P300 responses due to the addition of white noise. While one might argue that stochastic resonance can’t explain our pattern of results – as the FM modulation occurs at regular, foreseeable time intervals – the change of the FM modulation of the auditory stimulus occurs at a randomized point in time and can thus increase stochastic resonance. The alignment of the visual and auditory background distractors might have served a similar role as the noise additions in those experiments, explaining possible performance increases specifically for the audiovisual congruent condition.

In conclusion, a frequency alignment of FM modulated auditory and visual distractor stimuli led to an increase in accuracy when subjects performed an unrelated visual task, even at a different spatial location. A possible mechanism to explain our results might be an increase in stochastic resonance.

## Data Availability Statement

The datasets generated for this study are available on request to the corresponding author.

## Ethics Statement

Ethical review and approval was not required for the study on human participants in accordance with the local legislation and institutional requirements. The patients/participants provided their written informed consent to participate in this study. The study was conducted in accordance with the Declaration of Helsinki and approved by the ethics committee of the University of Leipzig.

## Author Contributions

All authors listed have made a substantial, direct and intellectual contribution to the work, and approved it for publication.

## Conflict of Interest

The authors declare that the research was conducted in the absence of any commercial or financial relationships that could be construed as a potential conflict of interest.
