# Supplementary material for: Alignment of Continuous Auditory and Visual Distractor Stimuli Is Leading to an Increased Performance
Source: Front Psychol. 2020 May 8;11:790. doi: 10.3389/fpsyg.2020.00790 (PMC7225351; doi:10.3389/fpsyg.2020.00790)
Supplement: Supplementary file 1 [file Data_Sheet_1.PDF]

## Supplemental Material

### *Participants*

A total of 24 participants (5 male, age range 19 – 32 years, 24 right handed) participated within the experiment. All participants had normal or corrected to normal hearing and vision, gave written and informed consent in accordance with the guidelines of the ethics committee of the University of Leipzig and with the Declaration of Helsinki and had been reimbursed with either course credits or 8€ per hour. The sample size of the experiment was calculated a priori, using G-Power (Erdfelder, Faul, & Buchner, 1996; Faul, Erdfelder, Lang, & Buchner, 2007), on the basis of the effect sizes from van der Burg et al. (2008). According to G-Power comparable significant behavioral modulations ($\alpha = 0.05$, $\eta^2=.68$) can be detected with 90% power using 24 Participants. One participant had to be discarded during the analysis due to an insufficient behavioral performance.

### *Methods*

An overview over the experiment is provided in Supplementary Figure 1. The experiment was similar to the main experiment except that the visual display consisted of a centrally presented fixation cross of two equally sized black lines with a length of 0.5° visual angle. One of the two arms of the fixation cross increased its length by 0.024° visual angle whenever an event was presented. The task was to detect a subtle increase in the length in one of the two arms of the fixation cross, presented centrally on the screen, and to discriminate if the event was presented vertically or horizontally. 50 % of all trials contained one or two events, with one event per event trial placed within one of ten equal sized time bins (from here on referred to as time windows) of 150 ms around the tone switch, starting from -150 ms (0 being the time of the switch) and ending at 1350 ms. Every time window contained the same number of targets over the course of the experiment. The events could onset at every frame of the time

windows, thus we decided to group all evens within the windows together and analyze the difference between the time windows. Half of the event trials contained a second event, presented in equal amount before (early) or after (late) the time windows with a minimum distance of 700 ms. In addition, the first 500 ms and the last 500 ms of each trial could not contain an event. The amount of early and late events was equalized and the order of all events pseudorandomized over the course of the experiment. The difficulty of the discrimination was adjusted to a performance of 80% in advance of the experiment by adjusting the duration of the event (average duration 138 ms). When detecting an event, participants had to press the left arrow key if the horizontal bar changed its length and the downward arrow key if the change was upon the vertical bar, which of the two bars would change was randomized.

### *Data Analysis*

The analysis was conducted similar to the main experiment, however, some participants showed large outliers in terms of accuracy (below 30%) and the outliers could only be removed as such by removing all datapoints differing 1.5 standard deviations from the mean. This affected less than 10% of all data and in order to avoid a reduction in statistical power the values were replaced with the condition mean.

## Results

### *Accuracy*

Participants performed the task with an average accuracy of 79.25% ± 0.12%. We found a main effect of condition ($F_{2.44,\ 53.77} = 3.660$, $p = .016$, $\eta^2 = .143$), as well as a main effect of time ($F_{2.97,\ 65,67} = 6.887$, $p < .001$, $\eta^2 = .426$) and a significant interaction between condition

and time ($F_{33, 726} = 1.528$, $p < .031$, $\eta^2 = .069$). The effects are displayed in Supplemental Figure 2. The main effect of condition revealed that the audiovisual congruent condition lead to an increased accuracy compared to the visual control condition (p = .034), and to the audiovisual incongruent condition (p = .034), but not against the auditory condition (p = .58). The main effect of time revealed that the accuracy within the late time window was significantly reduced in comparison to the accuracy in any other time window. The interaction between attention to time and modality (Suppl. Fig. 3) revealed a trend towards increased accuracy in the audiovisual congruent compared to the incongruent condition between 150-300ms (p = .064) and a significant decrease in accuracy for the audiovisual incongruent condition against the audiovisual congruent condition between 600 and 750 ms (p = .031). Both, however, were not significant against the baseline, potentially due to higher variability in the baseline conditions. Overall, we did find subtle differences between audiovisual congruent and incongruent condition and against the visual baseline condition, but not against the auditory baseline condition. Since the auditory condition remained insignificant against all other conditions (p = 1 against both the visual and audiovisual congruent condition), we decided to focus on the visual condition as control for further analysis.

### *Mean Reaction Times*

The mean reaction time across all conditions was 0.6383 s (*SD* = 0.06) and, similar to the accuracy results, we found a main effect of time ($F_{2.05, 45.16} = 4.486$, $p < .001$, $\eta^2 = .169$) and an interaction between condition and time ($F_{11.58, 260.15} = 2.033$, $p < .001$, $\eta^2 = .084$). The effect of condition was non-significant (p = 0.29).

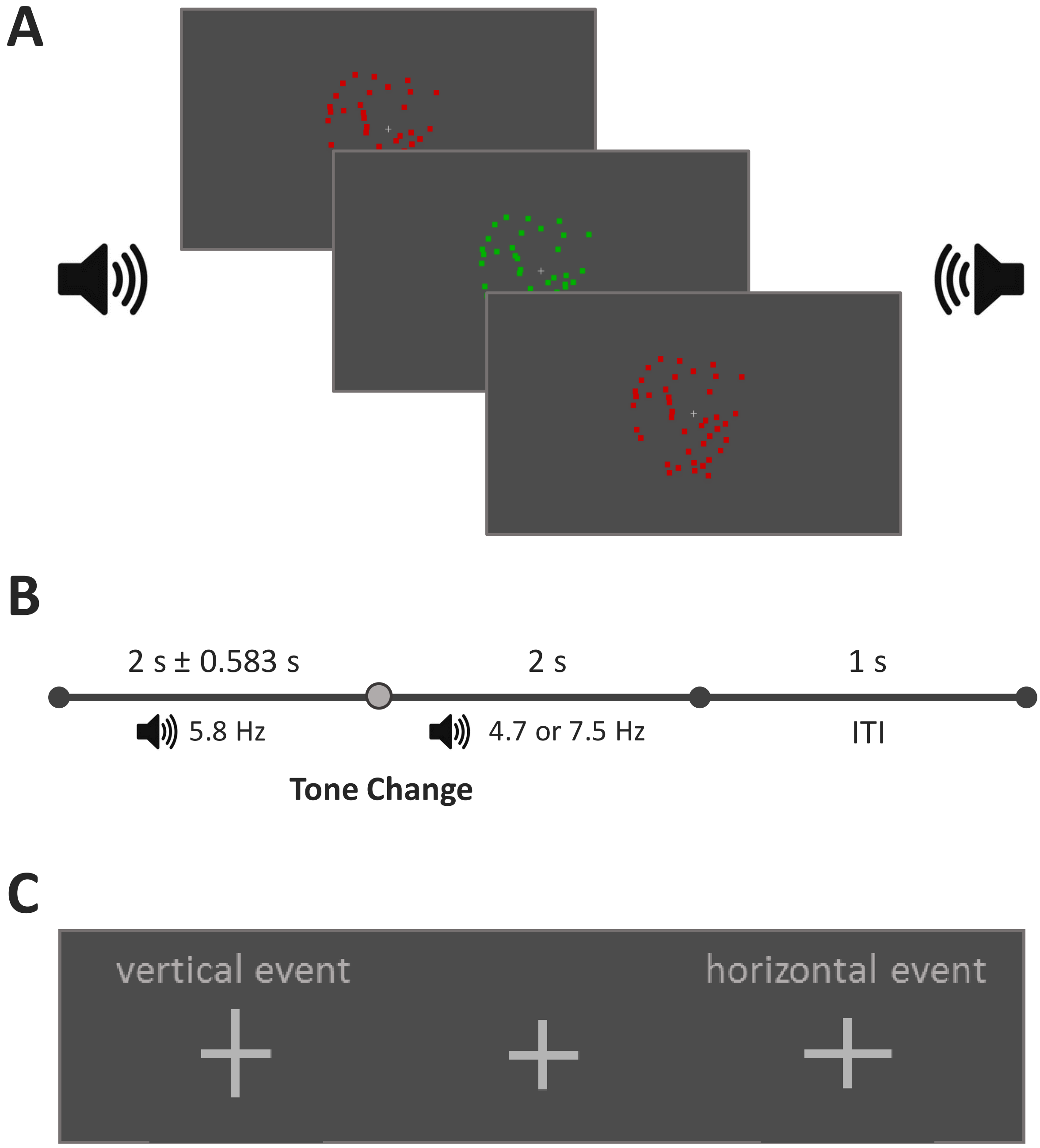


**Supplementary Figure 1:** Experiment Overview. (A) A fixation cross was presented on a grey screen, surrounded by a random dot kinematogram and a frequency modulated sound as distractors. (B) Timeline of the experiment. Within the audiovisual conditions and the auditory condition, a sound with a FM of 5.8 Hz would be presented at the start of the trial. After 2s ± a variable delay the sound would switch to an FM frequency of either 4.7 or 7.5 Hz and after a another 2s the visual and auditory distractor stimuli would vanish and the 1s

intertrial interval would start. (C) The task was to detect a subtle change of the fixation cross and to report which arm of the fixation cross had elongated.

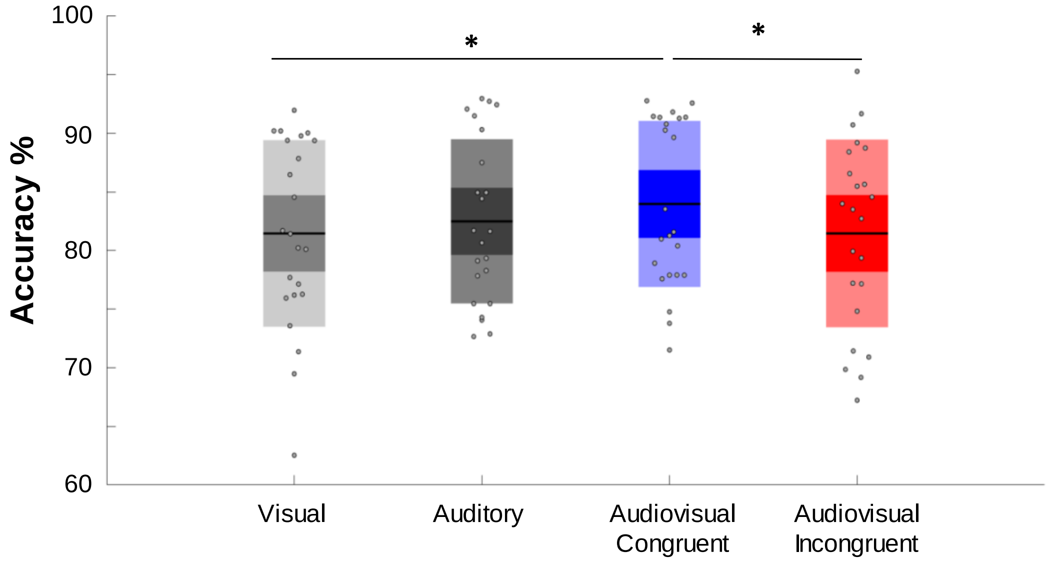


**Supplementary Figure 2:** Accuracy results of the pilot. Participants were significantly more accurate in audiovisual congruent trials than in visual or audiovisual incongruent trials. No difference towards the auditory condition was observed.

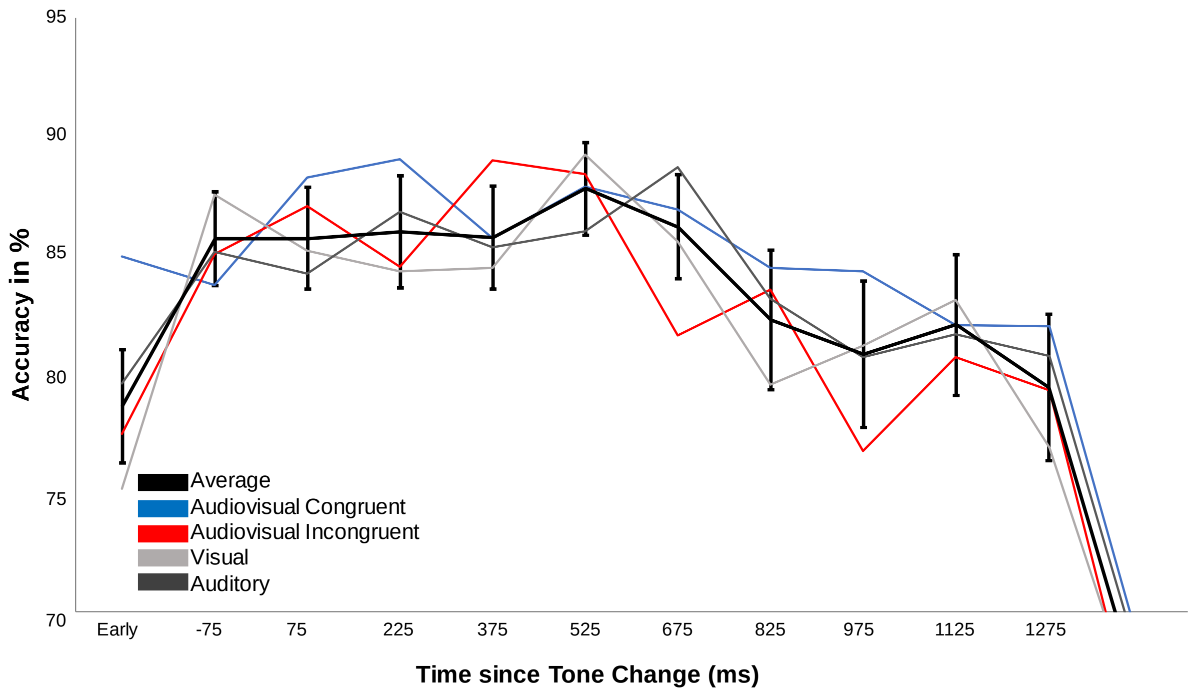


**Supplementary Figure 3:** Time-resolved accuracy results. The black line represents the average over all conditions, the blue and red line the congruent and incongruent conditions respectively, the light grey line the visual and the dark grey line the auditory control. Error bars display the standard error of the mean. Between 150 and 300 ms after the tone change the accuracy in the audiovisual congruent condition was marginally increased compared to the audiovisual incongruent condition, but did not significantly differ from the auditory or the visual control conditions. There was another significant difference between the audiovisual conditions, reflected by a drop in the accuracy of the audiovisual incongruent condition, 600 – 750 ms after the tone change, but again the change was not significant against the control conditions. While one can observe a significant drop in the overall accuracy for events appearing later than 1500 ms, no other significant difference between the different conditions at a specific point in time was observed.
